# Detroit Medical and Library Association

**Published:** 1877-11

**Authors:** 


### DETROIT MEDICAL AND LIBRARY ASSOCIATION.

Regular meeting September 24, 1877. President Brown in the chair.

Dr. McGraw exhibited a vesical calculus, lately removed, weighing 666 grains. The stone was of composite character, the nucleus being of uric acid, while around it were concentric layers of oxalic acid and various phosphates. The patient had been afflicted for years, but his malady had never been fully diagnosticated, probably on account of the extreme difficulty in reaching the stone, which lay sacculated above the pubis.

When the diagnosis was fully determined, the doctor operated by the medio-lateral method, with a straight staff, but was compelled to crush the stone with the lithotrite before he could extract it from the wound. The operation was a successful one, and the patient was nearly well, no urine passing by the perineal wound.

The case had come from outside the city, and this led to the remark that in his experience when a stone did not originate either from paralysis or enlarged prostate, it came from some locality outside of Detroit.

He also exhibited a large sarcomatous tumor, removed from the posterior triangle of the neck. It had involved the glands of that region, and entailed much suffering on the patient.

In its inception it had presented no symptoms of malignancy, which fact gave the doctor an opportunity to state his tendency to a belief in the theory that tumors are of local origin and benign in character, afterwards assuming malignancy. He also believed that early excision of tumors would prevent, by anticipation, this change in type, and thus preserve many an unfortunate from much suffering.

Dr. Jenks exhibited a tumor weighing seven and a half pounds, taken from the abdomen of a child two and a half years of age. He had first seen the child about ten months ago, with Dr. Leach, who was attending it for some malarial affection. His attention had been called by the mother to a bloody discharge, thought to be from the vagina, but which he now believes came from the bladder; and also to a "bloating" in the abdomen. An examination of the latter plainly revealed the presence of a tumor occupying entirely the left iliac fossa, rising above the umbilical line, and reaching beyond the median line. When Dr. Jenks examined the case he found the abdominal walls freely movable over the surface of the tumor, and fluctuation present.

It very much simulated an ovarian cyst, but a guarded diagnosis was given, as, if such were the fact, it would be a very unusual and remarkable thing.

Several other physicians also examined the child, and all inclined to the same opinion.

The tumor kept growing, the child failed, and recently died.

A post mortem was held, and on cutting down to the tumor, it was found to be encased in a thick, fibrous covering, and to be agglutinated to the intestines, the omentum and the mesentery. Along the spine, in the lumbar region, it was closely adherent, and being cut away from its attachments no kidney was found on that side. It was therefore a tumor of the left kidney. The right kidney, which had been doing the work of two, was healthy, and as large as an ordinary adult kidney. The tumor was then laid open in presence of the society, when its contents were so evidently encephaloid that no doubt was expressed as to its character.

Dr. McGraw then read a paper on "The Excision of the Inferior Dental and Gustatory Nerves, for the Cure of Dental Neuralgia."

Dr. Mulheron mentioned a patient he had recently seen on the eighth day after her confinement. She was then comatose and moribund from meningeal inflammation, but had never slept since her delivery. It was a question with him whether the sleeplessness was a cause or a result of the inflammation. By inquiry it was developed that the woman had seemed over-anxious about her household affairs, and had a sense of impending disaster, but no solution of the problem was offered.

Dr. McGraw said that within a few days he had been five miles into the country to see a case of typhoid fever, of which there had been several cases in the family or neighborhood. The cause of the disease could be very readily traced to the foulness of the water in the wells of the community. These cases had brought up with fresh interest the whole question of the miserable water supply of the farmers. It was also a question of vital importance to this city how far the butter and milk brought from the surrounding country might be contaminated with foul water, but it is a difficulty he now saw no way of surmounting.

Dr. Shurly said he had been attending a case of typhoid which, in its virulence, resembled the typhoid in the Eastern States. The patient was so reduced that all functions but the vegetative were extinct. He had expected other cases in the same family, but none occurred. He thought the books, especially English works, were too prone to consider typhoid fever purely contagious in character, while he believed there were endemic influences modifying the disease in many instances which were confounded with those of a contagious origin.

Dr. Heaton gave some account of typhoid fever as he knew it in the Lake Superior region, about Keewenaw Point.

On the north of the Point, he said, was the lake, and a little way back from the shore was a high bluff. A little further back, and over the ridge, lay the valley of Eagle river, covered with vast forests. When the mine owners began to clear these forests typhoid fever broke out with great virulence. The Cliff mine alone lost seventy-five men, the majority by hæmorrhage from the bowels. His observation led him to the belief that the fever resulted from a combination of causes, chief among which were the clearing of forests, the digging under old houses, or any enterprise which disturbed the accumulated decay of years or century.

Dr. Jenks spoke of the peculiarity of the water supply of Goderich, and also its remakable freedom from malarial diseases, though it was just across Lake Huron from Saginaw, where those diseases were very rife. They did have typhoid fever, however, and no one doubted that the following was the correct explanation of these facts:

The town lies on high ground, which rises rather steeply from the lake, and there is almost always a strong breeze blowing, which accounts for its freedom from malarial disease.

The supply of water was from wells, which had their source in a layer of gravel above a stratum of blue clay. In the town was a hollow, and it was in this neighborhood that the cases of typhoid occurred, and visitors were always cautioned against drinking from wells in that vicinity. There was no doubt that the water was contaminated by the surface drainage from the surrounding higher land of the city.

After some further desultory talk, Dr. Eugene Smith reported a case of punctured wound of the lens, the instrument being a sharp piece of small wire. The external wound was about a line from the corneo-sclerotic junction, whence the wire traversed the anterior chamber, and pierced the lens. When he saw the patient the aqueous humor had refilled the camber, though there was some blood mixed with it; still the wound in the lens could be seen. Dropped a solution of atropia into the eye, and applied a compress.

Next morning there was marked improvement, the blood being nearly all absorbed, though the lens was cloudy. The treatment was continued for two weeks, when recovery was complete, a most remarkable result, as cataract generally follows injuries of such character.

He also reported a case of enucleation of the eye for trachomatous degeneration. The disease had been called cancer, but he had no difficulty in making a diagnosis, as the neoplastic formation peculiar to trachoma was very marked. He made the operation on account of sympathetic irritation, and was obliged to extend the external angle of the eye by incision, the eyelids had become so contracted, which made the case one of unusual interest.—*Detroit Medical Journal.*

